# Epidemiological Characteristics and Underlying Risk Factors for Mortality during the Autumn 2009 Pandemic Wave in Mexico

**DOI:** 10.1371/journal.pone.0041069

**Published:** 2012-07-16

**Authors:** Gerardo Chowell, Santiago Echevarría-Zuno, Cécile Viboud, Lone Simonsen, Mark A. Miller, Irma Fernández-Gárate, Cesar González-Bonilla, Víctor H. Borja-Aburto

**Affiliations:** 1 Division of Epidemiology and Population Studies, Fogarty International Center, National Institutes of Health, Bethesda, Maryland, United States of America; 2 Mathematical, Computational & Modeling Sciences Center, School of Human Evolution and Social Change, Arizona State University, Tempe, Arizona, United States of America; 3 Dirección de Prestaciones Médicas, Instituto Mexicano del Seguro Social, Mexico City, México; 4 Department of Global Health, George Washington University School of Public Health and Health Services, Washington, D.C., United States of America; 5 Coordinación de Programas Integrados de Salud, Dirección de Prestaciones Médicas, Instituto Mexicano del Seguro Social, Mexico City, México; 6 Coordinación de Vigilancia Epidemiológica y Apoyo en Contingencias, Instituto Mexicano del Seguro Social, Mexico City, México; University of Hong Kong, Hong Kong

## Abstract

**Background:**

Elucidating the role of the underlying risk factors for severe outcomes of the 2009 A/H1N1 influenza pandemic could be crucial to define priority risk groups in resource-limited settings in future pandemics.

**Methods:**

We use individual-level clinical data on a large series of ARI (acute respiratory infection) hospitalizations from a prospective surveillance system of the Mexican Social Security medical system to analyze clinical features at presentation, admission delays, selected comorbidities and receipt of seasonal vaccine on the risk of A/H1N1-related death. We considered ARI hospitalizations and inpatient-deaths, and recorded demographic, geographic, and medical information on individual patients during August-December, 2009.

**Results:**

Seasonal influenza vaccination was associated with a reduced risk of death among A/H1N1 inpatients (OR = 0.43 (95% CI: 0.25, 0.74)) after adjustment for age, gender, geography, antiviral treatment, admission delays, comorbidities and medical conditions. However, this result should be interpreted with caution as it could have been affected by factors not directly measured in our study. Moreover, the effect of antiviral treatment against A/H1N1 inpatient death did not reach statistical significance (OR = 0.56 (95% CI: 0.29, 1.10)) probably because only 8.9% of A/H1N1 inpatients received antiviral treatment. Moreover, diabetes (OR = 1.6) and immune suppression (OR = 2.3) were statistically significant risk factors for death whereas asthmatic persons (OR = 0.3) or pregnant women (OR = 0.4) experienced a reduced fatality rate among A/H1N1 inpatients. We also observed an increased risk of death among A/H1N1 inpatients with admission delays >2 days after symptom onset (OR = 2.7). Similar associations were also observed for A/H1N1-negative inpatients.

**Conclusions:**

Geographical variation in identified medical risk factors including prevalence of diabetes and immune suppression may in part explain between-country differences in pandemic mortality burden. Furthermore, access to care including hospitalization without delay and antiviral treatment and are also important factors, as well as vaccination coverage with the 2008–09 trivalent inactivated influenza vaccine.

## Introduction

A number of researchers have explored the clinical and epidemiological characteristics of novel A/H1N1 influenza in different populations around the globe (e.g., [1], [2], [3], [4], [5], [6], [7], [8], [9], [10], [11], [12]), but analyses of large individual-level clinical data spanning multiple geographic regions and disease severity outcomes of A/H1N1 infections are scarce. These studies could be crucial to quantify the role of underlying population health, case management, hospital admission delays and potential changes in the influenza virus characteristics on the mortality burden of the 2009 A/H1N1 influenza pandemic across countries [13].

The 2009 A/H1N1 influenza pandemic virus spread heterogeneously throughout Mexico [14], [15], [16], [17], in a series of three pandemic waves in the spring, summer, and fall of 2009, which were associated with high mortality impact relative to other countries [13], [18], [19]. Although a preliminary analysis of the epidemiological characteristics and risk factors for A/H1N1 influenza infections has been carried out for the spring-summer pandemic period in Mexico [14], [15], [16], [20], a comprehensive analysis of the fall pandemic wave during which most of the deaths occurred, has not been carried out. Moreover, preliminary studies conducted in Mexico during the early pandemic phase suggested significant partial protection to novel 2009 A/H1N1 influenza conferred by the 2008–09 trivalent inactivated influenza vaccine (TIV) [20], [21]. Whether vaccination with the 2008–2009 seasonal influenza vaccine was significantly associated with a reduced risk of death in the fall has yet to be evaluated. In this article we fill this gap and carry out an analysis of clinical features at presentation, hospital admission delays, medical conditions, and receipt of seasonal vaccine on the risk of A/H1N1-related death among hospitalized patients. We use individual-level data from a prospective surveillance system implemented by the largest Mexican Social Security medical system spanning August-December, 2009.

## Materials and Methods

### Epidemiological Data

Individual level hospitalization data were available from a prospective epidemiological surveillance system implemented during the 2009 influenza pandemic by the Mexican Institute for Social Security (IMSS) [18], [20]. IMSS is a tripartite Mexican health system covering approximately 40% of the Mexican population comprising workers in the private sector and their families, relying on a network of 1,099 primary health-care units and 259 hospitals nationwide. The age and gender distributions of persons affiliated to the IMSS medical system are representative of the general Mexican population [18]. Respiratory swabs were obtained for approximately a third of cases with constant sampling intensity across states, time, and age groups [18]. Swabs were tested for pandemic A/H1N1 influenza virus by rRT-PCR by La Raza, an IMSS laboratory certified by the Instituto de Diagnóstico y Referencia Epidemiológica (InDRE).

We analyzed information from all hospitalizations and inpatient-deaths among patients admitted with acute respiratory infection (ARI) during the fall pandemic wave from August through December, 2009; the time period associated with widespread A/H1N1 activity particularly affecting northern and central Mexican states and coincided with the return of students from summer vacations [18]. ARI was defined as any person with respiratory difficulty presenting fever 38°C and cough, combined with one or more of the following clinical symptoms: confinement to bed, thoracic pain, polypnea, or acute respiratory distress syndrome. Children <5 years with pneumonia or severe pneumonia that required hospitalization were also considered as ARI cases.

For each ARI inpatient, we recorded demographic information (age in yrs, and gender), pandemic A/H1N1 status (positive, negative; for those tested by RT-PRC), reporting state (including 31 states plus the Federal District), clinical signs and symptoms at presentation (fever, cough, headache, sore throat, malaise, myalgia, rhinorrhoea, arthralgias, myalgia, prostration, thoracic pain, abdominal pain, conjunctivitis, nasal congestion, cyanosis, diarrhea, dyspnea, and coryza), underlying risk factors (chronic obstructive pulmonary disease (COPD), diabetes, smoking, obesity, pregnancy, lactating mother, immune status, asthma, and HIV/AIDS status), dates of onset of symptoms (self-reported) and hospitalization and discharge, whether the patient was treated with neuraminidase inhibitors, and the 2008–09 trivalent inactivated influenza vaccine (TIV) status. In Mexico, antiviral treatment with neuraminidase inhibitors (Oseltamivir and Zanamivir) was considered for patients upon initial clinical evaluation and individual risk of developing complications [22]. Specifically, antiviral treatment was recommended for all cases presenting severe symptoms, irrespective of age or underlying conditions. For cases presenting mild symptoms, antiviral treatment was recommended for high-risk patients only, which included infants <5 y, seniors >65 y, persons with lung disease (including asthma), cardiovascular disease (except for systemic arterial hypertension), renal disease, hematologic disease, neurologic disease, neuromuscular or metabolic disorders (including diabetes), and immune deficiency disease.

We defined the *admission delay* as the time elapsed from symptoms onset to hospitalization admission, and *hospital length of stay* was defined as the number of days from hospital admission to discharge or death. Given that recommendations for neuraminidase inhibitors stipulate that treatment should be provided within 48 h of disease onset, we stratified admission delay into two groups < = 2 and >2 days.

### Statistical Analysis

We used univariate and multivariate logistic regression models to assess the risk of death among A/H1N1-positive ARI inpatients. In particular we sought to investigate the effect of the 2008–2009 seasonal influenza vaccine status on the risk of death among A/H1N1-positive cases after adjustment for age, gender, geography, admission delay (< = 2 days vs. >2 days), antiviral treatment, and comorbidities. We also quantified the interaction between antiviral treatment and admission delay to account for the fact that patients admitted earlier in their disease course had a higher probability of receiving antiviral treatment. Because the 2009 pandemic vaccine was not available until December 2009 in Mexico [23], we did not seek to include pandemic vaccination status in the model. The effects of model predictors were measured using odds ratios (95% CI) and P values. Records with missing data (e.g., admission delay, comorbidities) were excluded from the analysis.

Observational studies like ours are prone to confounding bias due to their inherent observational nature [24]. To assess confounding bias, we compared our findings based on A/H1N1-positive inpatients with those based on A/H1N1-negative inpatients. Specifically, we analyzed the risk of death in the group of A/H1N1-negative inpatients using multivariate logistic regression after adjustment for age, gender, geography, admission delays (< = 2 days vs. >2 days), antiviral treatment, the 2008–2009 seasonal influenza vaccine status, and underlying comorbidities and other medical conditions.

Ethics Committee approval was not necessary according to local regulations as all the data were de-identified and information used in this study is routinely collected for epidemiological surveillance purposes.

Statistical analyses were performed using PASW Statistics 18.0.

## Results

### Hospital admission delay and length of stay

**Figure 1:**
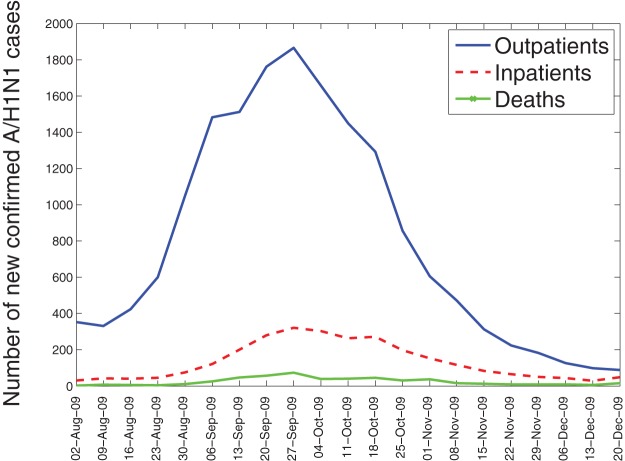
Weekly number of new laboratory-confirmed A/H1N1 influenza outpatients, inpatients, and deaths by dates of symptoms onset, August-December 2009 in Mexico.

Out of the 10435 ARI hospitalizations recorded during the fall pandemic wave, the number of A/H1N1-positive hospitalizations and inpatient deaths were 2944 and 517, respectively (Figure 1). The case fatality proportion was significantly higher among male A/H1N1 inpatients compared to females (17.3% vs. 13.1%, Chi-square test, P = 0.001). The great majority of laboratory-confirmed influenza inpatients were 18–49 y (51.7%) followed by persons <18 y (31%) and those >50 y (17.3%). The case fatality proportion increased with older age from 7.4% for persons <18 y, 16.6% for persons 18–49 y, and 23.5% for persons > = 50 y.

The average time from symptoms onset to admission among A/H1N1-positive inpatients was 3.1 days (95% CI: 3.0, 3.3) while the average length of hospital stay was 5.3 days (95% CI: 5.1, 5.6), but it was significantly longer for A/H1N1 inpatients with fatal outcomes than for those who recovered (4.9 days (95% CI: 4.7, 5.1) vs. 7.7 (95% CI: 7.0, 8.4), Wilcoxon test, P<0.0001). Among A/H1N1 inpatients that recovered, the length of hospital stay was 4.1 days (95% CI: 3.9, 4.3) among inpatients with admission delays < = 2 days compared to inpatients with admission delays >2 days (5.8 days (95% CI: 5.4, 6.2), Wilcoxon test, P<0.0001).

### Clinical symptoms

The most common signs and symptoms among A/H1N1-positive inpatients were cough, malaise, headache, and fever as shown in Table 1. Dyspnea, cyanosis, and prostration at presentation were significantly associated with the risk of death among A/H1N1-positive inpatients after adjustment for age, gender, and geography using a multivariate logistic regression framework (Table 1). For comparison, dyspnea, cyanosis, and thoracic pain were statistically significant risk factors of death among A/H1N1-negative inpatients, but presence of prostration was not statistically significant.

**Table 1:** Frequency of clinical characteristics of laboratory-confirmed A/H1N1inpatients and fatal cases, Mexico, August-December 2009.

Clinical features	A/H1N1 hospitalizations for surviving patients	A/H1N1 deaths	A/H1N1-inpatient vs A/H1N1 death odds ratio (95% CI)^a^
Fever	2365/2940 (80.4)	397/516 (76.9)	0.95 (0.73, 1.24)
Cough	2728/2940 (92.8)	474/516 (91.9)	0.96 (0.65, 1.42)
Headache	2462/2940 (83.7)	387/516 (75)	0.82 (0.62, 1.08)
Sore throat	1686/2940 (57.3)	257/516 (49.8)	0.86 (0.69, 1.07)
Malaise	2536/2940 (86.3)	439/516 (85.1)	1.07 (0.78, 1.49)
Myalgia	2156/2940 (73.3)	331/516 (64.1)	0.80 (0.56, 1.15)
Arthralgia	2059/2940 (70)	315/516 (61)	0.97 (0.68, 1.38)
Prostration	1676/2940 (57)	334/516 (64.7)	1.38 (1.10, 1.75)
Rhinorrhea	2055/2940 (69.9)	264/516 (51.2)	0.53 (0.43, 0.66)
Chills	1942/2940 (66.1)	289/516 (56)	0.73 (0.58, 0.93)
Abdominal pain	865/2940 (29.4)	141/516 (27.3)	0.92 (0.72, 1.17)
Conjuntivitis	973/2940 (33.1)	100/516 (19.4)	0.53 (0.41, 0.69)
Dyspnea	1559/2940 (53)	406/516 (78.7)	2.63 (2.04, 3.40)
Cyanosis	205/2940 (7)	148/516 (28.7)	4.58 (3.50, 6.01)
Diarrhea	371/2940 (12.6)	82/516 (15.9)	1.17 (0.87, 1.57)
Thoracic pain	1425/2940 (48.5)	271/516 (52.5)	0.95 (0.75, 1.19)
Coryza	354/2940 (12)	42/516 (8.1)	0.64 (0.43, 0.93)

a adjusted by age, gender, and geography.

### Underlying risk factors

**Figure 2:**
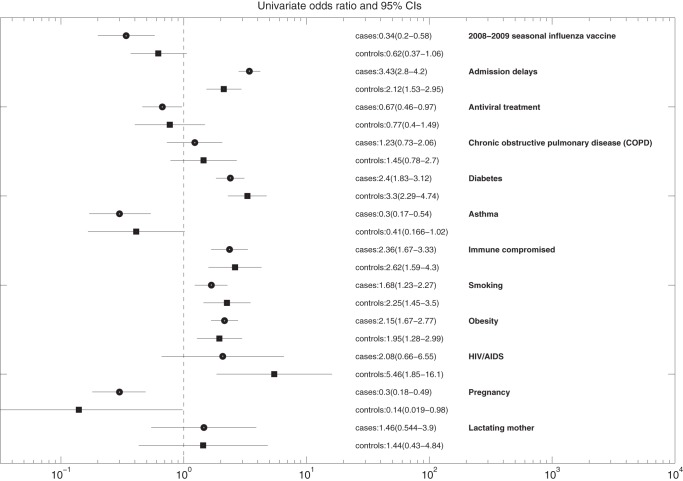
Results of univariate logistic regression analyses of the risk of death among inpatients based on A/H1N1-positive inpatients and A/H1N1-negative inpatients (controls). The effects of model predictors were measured using odds ratios and corresponding 95% CIs.

**Figure 3:**
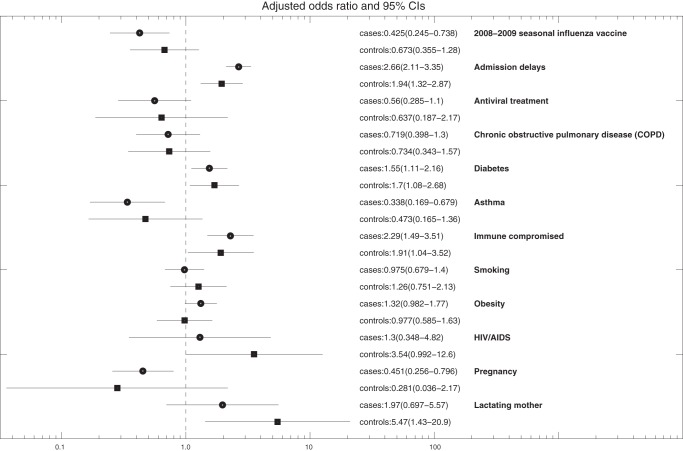
Results of a multivariate logistic regression analysis of the risk of death among inpatients based on A/H1N1-positive inpatients and A/H1N1-negative inpatients (controls) after adjusting for age, gender, geography, antiviral treatment, admission delays, 2008–2009 seasonal influenza vaccine, comorbidities, and other medical conditions. The effects of model predictors were measured using odds ratios and corresponding 95% CIs.

A comparison of the characteristics of A/H1N1-postive and A/H1N1-negative inpatients including age, gender, geography, comorbidities, antiviral treatment, admission delays, and 2008–2009 seasonal influenza vaccine status is shown in Table 2. Among inpatients with confirmed A/H1N1, one or more comorbidities were present in 16.5% of A/H1N1 inpatients under 18 years of age and in 32.2% of inpatients 18 years and older. The most common comorbidities among A/H1N1 inpatients were obesity (11.8%) and diabetes (9.1%) (Table 3). In univariate logistic regression analyses, diabetes, immune suppression, smoking and obesity were significantly associated with an increase risk of death among A/H1N1-positive inpatients while asthma and pregnancy were significantly associated with a reduced risk of death among A/H1N1-positive inpatients (Figure 2). Similar effects were observed in our comparison group of A/H1N1-negative inpatients (Figure 2). In an adjusted multivariate logistic regression analysis of the risk factors for dying from A/H1N1 influenza ARI hospitalization, we found diabetes (OR = 1.55 (95% CI: 1.11, 2.16)) and immune suppression (OR = 2.29 (95% CI: 1.49, 3.51)) to be statistically significant risk factors while asthma (OR = 0.34 (95% CI: 0.17, 0.68)) and pregnancy (OR = 0.43 (95% CI: 0.25, 0.74)) were significantly associated with a reduced risk of death among A/H1N1 inpatients with adjustment for age, gender, geography, admission delay, antiviral treatment, and 2008–2009 seasonal influenza vaccine status (Table 3). These risk factors had a similar effect in our group of A/H1N1-negative inpatients (Figure 3).

**Table 2:** Characteristics of A/H1N1-positive and A/H1N1-negative inpatients and all ARI inpatients by age, gender, geography, 2008–2009 seasonal influenza vaccine status, and comorbidities and other medical conditions, Mexico, August-December 2009.

Variable	A/H1N1-positive inpatients	A/H1N1-negative inpatients		All ARI inpatients	
**Geographic region**					
Central	1757 (50.8%)	1128 (50.3%)		4871 (46.7%)	
Southern	375 (10.8%)	350 (15.6%)		1363 (13.1%)	
Other states	1329 (38.4%)	766 (34.1%)		4197 (40.2%)	
**Demography**					
Female	1974 (57.1%)		1159 (51.9%)		5703 (54.9%)
Male	1482 (42.9%)		1073 (48.1%)		4685 (45.1%)
**Age (years)**					
0–4	386 (11.2%)	674 (40.4%)		2055 (19.8%)	
5–14	531 (15.4%)	191 (8.6%)		1271 (12.3%)	
15–29	972 (28.1%)	334 (15%)		2208 (21.3%)	
30–44	737 (21.3%)	420 (18.9%)		2145 (20.7%)	
45–59	616 (17.8%)	336 (15.1%)		1731 (16.7%)	
> = 60	212 (6.1%)	265 (11.9%)		961 (9.3%)	
**Admission delay >2 days**	1396 (42%)	1144 (53.4%)		4779 (47.9%)	
**Antiviral treatment**	306 (8.9%)	145 (6.5%)		768 (7.4%)	
**Comorbidities & medical conditions**					
Chronic obstructive pulmonary disease (COPD)	102 (3%)	102 (4.6%)		385 (3.7%)	
Diabetes	313 (9.1%)	231 (10.4%)		959 (9.3%)	
Asthma	227 (6.6%)	130 (5.9%)		680 (6.6%)	
Immune compromised	174 (5.1%)	113 (5.1%)		513 (5%)	
Smoking	274 (8%)	166 (7.5%)		766 (7.5%)	
Obesity	383 (11.8%)	204 (9.3%)		1015 (10.3%)	
HIV/AIDS	15 (0.4%)	15 (0.7%)		58 (0.6%)	
Pregnancy	320 (9.3%)	77 (3.5%)		613 (6%)	
Lactating mother	25 (0.8%)	26 (1.2%)		94 (1%)	
**2008–2009 TIV vaccinated**					
Total	253 (7.3%)	272 (12.2%)		964 (9.3%)	
Age group					
0–4	44 (17.4%)	128 (47.1%)		341 (35.4%)	
5–14	32 (12.6%)	13 (4.8%)		72 (7.5%)	
15–29	53 (20.9%)	23 (8.5%)		123 (12.8%)	
30–44	41 (16.2%)	42 (15.4%)		145 (15%)	
45–59	57 (22.5%)	37 (13.6%)		147 (15.2%)	
> = 60	26 (10.3%)	29 (10.7%)		136 (14.1%)	

**Table 3:** Frequency of risk factors and adjusted odds ratios for risk of death among laboratory-confirmed A/H1N1 inpatients, Mexico, August-December 2009.

Risk factors	All A/H1N1 Inpatients	A/H1N1 Fatal Inpatients	A/H1N1-hospitalization vs A/H1N1 death odds ratio (95% CI)^a^
Chronic obstructive pulmonary disease (COPD)	102/3426 (3)	18/512 (3.5)	0.72 (0.40, 1.30)
Diabetes	313/3426 (9.1)	86/512 (16.8)	1.55 (1.11, 2.16)
Asthma	227/3426 (6.6)	12/512 (2.3)	0.34 (0.17, 0.68)
Immune compromised	174/3426 (5.1)	49/512 (9.6)	2.29 (1.49, 3.51)
Smoking	274/3426 (8)	60/512 (11.7)	0.98 (0.68, 1.40)
Obesity	383/3259 (11.8)	99/500 (19.8)	1.32 (0.98, 1.78)
HIV/AIDS	15/3426 (0.4)	4/512 (0.8)	1.30 (0.35, 4.82)
Pregnancy^b^	302/1156 (26.1)	17/512 (3.3)	0.43 (0.25, 0.74)
Lactating mother	25/3323 (0.8)	5/488 (1)	1.97 (0.70, 5.57)

a Adjusted by age, gender, geography, admission delay, antiviral treatment, and 2008–2009 seasonal influenza vaccine status.

b Pregnant case denominators include all female patients of childbearing age (15–49 y).

A total of 1156 A/H1N1 influenza hospitalizations were among women of childbearing age (15–49 y) of which 26.1% were pregnant, 12.5% received antiviral medication and 7.2% reported receipt of the 2008–2009 seasonal influenza vaccine. Among the 320 hospitalized pregnant women with A/H1N1, 10.9% received antiviral medications and 8.4% had received the 2008–2009 seasonal influenza vaccine. The case fatality proportion among A/H1N1 pregnant women was significantly lower than that of non-pregnant women of childbearing age (5.3% vs. 15.3%, Chi-square test, P<0.0001). Table S1 shows the risk for death for pregnant women among women aged 15–49 years, stratified into 5-year age groups, hospitalized with laboratory-confirmed A/H1N1 influenza. The average hospital length of stay was not significantly different between pregnant women and non-pregnant woman of childbearing age that recovered (4.5 d vs. 4.7 d, t-test, P = 0.57).

### 2008–2009 seasonal influenza vaccine status

A total of 253 (7.3%) A/H1N1-positive inpatients reported receipt of the 2008–2009 seasonal influenza vaccine while 274 (12.2%) of A/H1N1-negative inpatients reported receipt of the seasonal vaccine. After adjusting for age, gender, geography, and treatment with neuraminidase inhibitors in a multivariate logistic regression framework, we found that immunization with the 2008–2009 seasonal influenza vaccine was significantly protective against death among A/H1N1 inpatients (OR = 0.32 (95% CI: 0.19, 0.55)). This significant protective effect was maintained even after further adjustment for admission delays and underlying comorbidities and other medical conditions (OR = 0.43 (95% CI: 0.24, 0.74), Figure 3). By contrast, receipt of the seasonal influenza vaccine by A/H1N1-negative inpatients was not statistically associated with risk of death (OR = 0.67 95% CI: 0.35, 1.128)). When this analysis was focused on the highest A/H1N1 incidence month of October, we found consistent results with estimated OR = 0.34 (95% CI: 0.12, 0.98). In contrast, for the low incidence month of August, we found non –significant estimates 0.25 (95% CI: 0.02, 2.7).

### Antiviral treatment and admission delays

Only 8.9% of A/H1N1-positive inpatients and 6.5% of A/H1N1-negative inpatients were treated with neuraminidase inhibitors during the 2009 fall pandemic wave in Mexico. Antiviral treatment was found to be a significant protective factor against death given A/H1N1 hospitalization (OR = 0.64 (95% CI: 0.43, 0.93)) after adjustment for age, gender, geography, and 2008–2009 seasonal influenza vaccine status. However, it was not statistically significant after further adjustment for admission delay and underlying medical conditions (OR = 0.56 (95% CI: 0.29, 1.10), Figure 3) perhaps due to the small fraction of inpatients treated with antivirals in the fall while admission delays >2 days significantly increased the risk of death among A/H1N1 inpatients (OR = 2.66 (95% CI: 2.11, 3.35)). Antiviral treatment was not statistically significant for the group of A/H1N1 inpatients with admission delays < = 2 days (OR = 0.52 (95% CI: 0.26, 1.04)) and for the group with admission delays >2 days (OR = 0.949 (95% CI: 0.56, 1.60)) after adjustment for all other covariates. We note that admission delays >2 days were also significantly associated with an increased risk of death among A/H1N1-negative inpatients (OR = 1.94 (95% CI: 1.32, 2.87)) after adjustment for other covariates.

## Discussion

In this study we have analyzed the clinical features, risk factors, and the effect of the 2008–09 trivalent inactivated influenza vaccine status on the risk of death using a large series of 10435 ARI inpatients during the fall pandemic wave (August – December, 2009) associated with most pandemic deaths in Mexico. Our results indicate that prior vaccination with the 2008–2009 seasonal influenza vaccine was significantly associated with a reduced risk of death among laboratory-confirmed A/H1N1 inpatients after adjustment for demographic and geographic information, comorbidities and other medical conditions, antiviral treatment, and admission delays using multivariate logistic regression. Nevertheless, this protective vaccine effect needs to be interpreted with caution as it could have been affected by factors not directly measured in our study (e.g., healthy vaccinee effect [25], [26]). Our findings also underscore the important role of underlying medical conditions such as diabetes and immune suppression and hospital admission delays in increasing the risk of death in patients hospitalized with A/H1N1.

Studies of the cross-reactive antibody response to a novel influenza A (H1N1) virus after vaccination with the 2008–2009 seasonal influenza vaccine indicated a small or no increase in antibodies against the novel 2009 swine-origin A/H1N1 influenza virus [27], [28], [29]. However, it was unknown whether such levels of cross-reactive antibody were able to confer any protection against novel influenza A/H1N1. In addition, the results of immunoinformatic studies that compared T-cell epitopes contained in novel 2009 A/H1N1 virus with epitopes in 2008–2009 conventional influenza vaccine suggested certain degree of protection against novel A/H1N1 [30], [31]. Furthermore, T cell-mediated immunity after influenza vaccination or natural infection has been shown to play an important role in heterotypic immunity [32], [33], [34]. From October 2008 to March 2009, approximately 14% of the IMSS-affiliated population were vaccinated with 2008–09 trivalent inactivated influenza vaccine (TIV) (79% among infants <3 years and 61.7% among seniors > = 60 years) [35]. Besides, reactive vaccination with the 2008-200 TIV seasonal vaccine was conducted in Mexico during the early 2009 pandemic period that included the administration of 1 million vaccine doses in the Federal District, but we were unable to ascertain cumulative seasonal influenza vaccination rates prior to the fall pandemic wave. Our results indicate that A/H1N1 inpatients vaccinated with the 2008–2009 seasonal influenza vaccine had a significant reduced risk of death (adjusted OR = 0.43; after controlling for age, gender, geography, comorbidities, antiviral treatment, and admission delay). We note that two earlier observational studies found a similar protective effect, based on a substantially smaller sample size than used here from the first months of the pandemic (April – July, 2009) in Mexico. These studies suggested a significant protective effect of 35%–73% against infection, particularly severe forms of the disease during the early pandemic phase [20], [21]. By contrast, other epidemiological studies have found no association [24], [36], [37], [38], [39], [40], [41], [42] or a mild benefit [43], [44] against infection or hospitalization with laboratory-confirmed pandemic A/H1N1 from the 2008–2009 seasonal influenza vaccine. However, these studies did not quantify the effect of seasonal influenza vaccine against death associated with pandemic A/H1N1 infection. By contrast, Canadian studies using data from the provinces of Alberta, British Colombia, Ontario, and Quebec [45], [46] reported the opposite effect with seasonal influenza vaccination in the previous season, with a significantly associated increased risk of A/H1N1 illness by 1.03- to 2.74-fold, after careful adjustment for comorbidities, age, and geography although this risk did not extend to hospitalized patients [47].

Risk of death among A/H1N1 influenza inpatients increased by a factor of 2.66 (95% CI: 2.11, 3.35) with admission delays >2 days after adjusting for other covariates, which is in line with previous studies [48], [49], [50], [51]. Overall the distribution of admission delays in our study is similar to that reported in other studies [50], [52], [53], [54]. This finding suggests that late access to medical care could explain an important fraction of the 2009 A/H1N1 influenza pandemic mortality burden in Mexico.

We identified some symptoms – dyspnea, cyanosis, and prostration – at presentation that were statistically significant predictors of death among A/H1N1-positive inpatients after adjusting by age and gender, in agreement with three previous reports [20], [55], [56].

Presence of diabetes (OR = 1.55 (95% CI: 1.11, 2.16)) and immune suppression (2.29 (95% CI: 1.49, 3.51)) were statistically significant risk factors of mortality among A/H1N1 inpatients in an adjusted multivariate logistic regression analysis. A similar effect was observed in the group of A/H1N1-negative inpatients (Figure 3). Of note, a national survey conducted among IMSS affiliated population in 2010 indicated that 6.2% of persons aged 20–59 years and 28.1% of persons older than 60 years had been previously diagnosed with diabetes mellitus. Furthermore, a retrospective study found diabetes and class III obesity to be significantly associated with death from pandemic A/H1N1 influenza in Southern Brazil [57]. In our study obesity was not a significant risk factor after controlling for diabetes and other underlying medical conditions using our multivariate logistic regression modeling framework applied to individual-level clinical data. In our data, 9.1% of A/H1N1 inpatients had diabetes, 11.8% of A/H1N1 inpatients were obese, and 2.6% of A/H1N1 inpatients were obese and diabetic. Several studies have supported a link between obesity and increased risk of death with seasonal influenza [58] and 2009 pandemic A/H1N1 influenza [8], [10], [11], [43], [57], [59], [60]. Of note, Morgan et al. [8] found morbid obesity to be significantly associated with 2009 A/H1N1 influenza severe outcomes, and Yu et al. [3] found obesity to be a risk factor among A/H1N1 hospitalizations in China.

Pregnancy was significantly associated with a reduced risk of death among A/H1N1 inpatients (OR = 0.43 (95% CI: 0.25, 0.74)) in a multivariate logistic regression framework after adjusting for admission delay and other covariates. A similar effect was observed in the group of A/H1N1-negative inpatients although it was not significant. It is possible that this apparently paradoxical finding can be explained by a tendency to hospitalize pregnant women for less severe influenza than age peers. In our sample, pregnant women accounted for 26% of A/H1N1 influenza hospitalizations, which is in agreement with Trulove et al. [61] who reported 27% of A/H1N1 hospitalizations among women of childbearing age in Wisconsin. However, these estimates are higher than the estimate of 19.6% reported for Canada [62]. Variations in the association of pregnancy and disease severity from A/H1N1 infections in different countries have been attributed to differences in case management practices [10], [11], [20], [63], [64].

We found asthmatic inpatients to be significantly associated with a reduced risk of death among A/H1N1 inpatients (OR = 0.34 (95% CI: 0.17, 0.68)), which is in line with a multi-country risk factor analysis of severe outcomes associated with pandemic A/H1N1 infections [10]. Van Kerkhove et al. [10] found that a higher proportion of hospitalized cases with asthma survived compared to patients with other conditions. These researchers explained this observation as the result of manageable influenza-induced exacerbations of asthma prompting admission that do not progress to viral pneumonia or other fatal complications. Another study by O'Riordan et al. found asthma to be a risk factor for severe disease among hospitalized children, but there was no clear relationship with severity of asthma [6], and Ward et al. [11] found asthma requiring regular preventive medication to be associated with hospitalization from A/H1N1 influenza.

Treatment with antivirals during the fall pandemic wave was not significantly associated with the risk of death among A/H1N1 inpatients after adjustment for admission delay and comorbidities and other medical conditions. This result could be due to the small fraction of hospitalized patients that were treated with antivirals during the fall wave (∼9%) and the fact that decision to treat was not found to be related to symptoms severity [65]. We [65] recently reported a sharp drop in antiviral use from 50% in the spring and summer wave to 9% in the main fall pandemic in Mexico. In addition, underlying confounding bias could have played a role, such as delayed patients being more likely to die and also being outside the “time window” of 48 hours when treatment with neuraminidase inhibitors is considered clinically meaningful [66], [67].

Our study has several strengths and limitations worth noting. We used data on a large series of ARI hospitalizations reported to the largest Mexican Social Security medical system where about one-third of cases were consistently tested for influenza over time and across age groups and geographic regions, and we have documented no evidence of weaker disease surveillance in smaller states [18]. Our individual-level clinical data allowed us to assess the effect of the 2008–2009 seasonal influenza vaccine status on severe disease outcomes associated with novel A/H1N1 influenza after controlling for demographic and geographic information, comorbidities and other medical conditions, antiviral treatment and hospital admission delays. Moreover, risk factor data were recorded in most medical records with only obesity information missing in 5.8% of inpatient records and other comorbidities missing in 1%. Admission delay was missing in 4% of records, and seasonal influenza vaccine status or antiviral treatment was missing in 0.1% of records. We were not able to ascertain differences in availability of critical case management and intensive care units by geographical region. Some comorbidities namely cardiovascular disease, renal disease, neurologic disorders, hematologic disease and hepatic conditions were not recorded, but have been found to be associated with severe disease from influenza infection [68], [69], [70], [71], [72]. Hence, we cannot rule out residual confounding bias in our study. Moreover, protective effects we observed against death might be related to confounding by factors that were not measured in our study such as the possibility that individuals who are vaccinated tend to be healthier than individuals who are not vaccinated (e.g., healthy vaccinee effect [25], [26]). The fact that the vaccine effect remained significant when the analysis was restricted to the peak month of influenza circulation in October, but disappeared when the analysis was restricted to August when influenza circulation was low, suggests however that this bias is limited.

We note that observational studies like ours are prone to confounding bias due to their inherent observational nature. Of note, study patients, who visited the IMSS facilities, were likely to be representative of those patients with more severe disease, given the representativeness of the IMSS system. Although the IMSS population is representative of the age and gender of the general Mexican population, our findings may not be representative of the lower socio-economic sector which is not covered by IMSS. Lastly, a subset of our A/H1N1-positive inpatients may have had secondary bacterial infection, but co-infection data was not available to evaluate the role of bacterial coinfections or antibiotic treatment on risk of death associated with A/H1N1 infection [73], [74].

In summary, our findings suggest that the 2008–9 trivalent inactivated vaccine was significantly associated with a reduced risk of death from 2009 A/H1N1 influenza infection during the fall pandemic wave in Mexico. Our study also highlights the role of admission delays, and prevalence of diabetes and immune suppression in explaining between-individual variation in 2009 A/H1N1 mortality risk, thereby potentially accounting for geographical variation in pandemic mortality rates across countries.

## Supporting Information

Table S1 Risk for death for pregnant and non-pregnant women aged 15–49 years, stratified into 5-year age groups, hospitalized with laboratory-confirmed A/H1N1 influenza in Mexico, August-December 2009.
(DOC)
