# Supplementary material for: Epidemiological Characteristics and Underlying Risk Factors for Mortality during the Autumn 2009 Pandemic Wave in Mexico
Source: PLoS One. 2012 Jul 16;7(7):e41069. doi: 10.1371/journal.pone.0041069 (PMC3397937; doi:10.1371/journal.pone.0041069)
Supplement: Table S1 — Risk for death for pregnant and non-pregnant women aged 15–49 years, stratified into 5-year age groups, hospitalized with laboratory-confirmed A/H1N1 influenza in Mexico, August-December 2009. (DOC) [file pone.0041069.s001.doc]

**Table S1.** Risk for death for pregnant and non-pregnant women aged 15-49 years, stratified into 5-year age groups, hospitalized with laboratory-confirmed A/H1N1 influenza in Mexico, August-December 2009.

| **Age group** |  | **Hospitalized non-pregnant women** | **Hospitalized pregnant women** | **Odds ratio for mortality (95% CI)** |
| --- | --- | --- | --- | --- |
| 15-19 | **Recovered** | 87 | 42 | 0.38 (0.08, 1.78) |
|  | **Death** | 11 | 2 |  |
| 20-24 | **Recovered** | 104 | 88 | 0.46 (0.18, 1.15) |
|  | **Death** | 18 | 7 |  |
| 25-29 | **Recovered** | 144 | 99 | 0.31 (0.1, 0.93) |
|  | **Death** | 19 | 4 |  |
| 30-34 | **Recovered** | 100 | 30 | 0.71 (0.19, 2.65) |
|  | **Death** | 14 | 3 |  |
| 35-39 | **Recovered** | 89 | 23 |  |
|  | **Death** | 31 | 0 |  |
| 40-44 | **Recovered** | 87 | 4 |  |
|  | **Death** | 19 | 0 |  |
| 45-49 | **Recovered** | 112 | 0 |  |
|  | **Death** | 19 | 0 |  |

^a^ Adjusted by age, gender, geography, admission delay, antiviral treatment, and 2008-2009 seasonal influenza vaccine status

^b^ Pregnant case denominators include all female patients of childbearing age (15-49 y)
